# A Case of Toxic Epidermal Necrolysis Caused by the Use of Trimethoprim Alone

**DOI:** 10.7759/cureus.14783

**Published:** 2021-04-30

**Authors:** Mala Sood, Samson O Oyibo, Jeyanthy Rajkanna

**Affiliations:** 1 General Medicine, Peterborough City Hospital, Peterborough, GBR; 2 Diabetes and Endocrinology, Peterborough City Hospital, Peterborough, GBR

**Keywords:** multidisciplinary team, stevens-johnson syndrome (sjs), trimethoprim, toxic epidermal necrolysis, ten, blistering, body surface area, supportive care, skin biopsy

## Abstract

Toxic epidermal necrolysis (TEN) is a rare, acute, severe mucocutaneous reaction commonly presenting following medication use. Thorough history taking and clinical examination are key to early diagnosis and management; skin biopsy provides diagnostic confirmation. We present a 54-year-old man who developed a widespread erythematous rash soon after the use of trimethoprim for an episode of acute prostatitis. An initial diagnosis of Stevens-Johnson syndrome evolved into toxic epidermal necrolysis following the rapid progression of his condition to a severe, blistering, and desquamating rash affecting more than 60% of his body surface area and mucosa. Through careful management with best supportive care and clinical judgement regarding the role of pharmacological intervention, he made a steady recovery supported by the wider multidisciplinary team. This is one of the very few reports in the literature implicating trimethoprim alone as an etiological agent in a severe case of TEN.

## Introduction

Stevens-Johnson syndrome (SJS) and toxic epidermal necrolysis (TEN) are severe mucocutaneous reactions, usually secondary to drugs, characterized by severe burn-like blistering and epithelial sloughing. Although rare, SJS and TEN are devastating diseases with high mortality in the case of TEN due to secondary systemic infection and multi-organ failure [1]. Current United Kingdom guidelines exist for the management of SJS and TEN and provide direction with regards to the history taking, diagnosis, care setting, multidisciplinary approach to managing each system involved, and appropriate follow-up [2].

Trimethoprim-sulfamethoxazole antibiotics are a well-established cause for SJS and TEN; however, limited case reports exist regarding trimethoprim as a single precipitating etiological agent. We present a case of severe life-threatening TEN secondary to trimethoprim and the journey to recovery.

## Case presentation

Medical history and demographics

A 54-year-old man presented to the emergency department with a generalized skin rash. He reported waking up with numb lips, bloodshot eyes, and epigastric discomfort, followed by a sore mouth, painful swallowing, and a generalized burning sensation of his skin. Two weeks prior to presentation, he was seen by his general practitioner for symptoms of dysuria and poor urinary flow for which he was given a 2-week course of trimethoprim for acute prostatitis. These symptoms resolved; however, he soon developed a headache and generalized muscle aches for two days before the rash appeared. His past medical history included acne rosacea for which he had been taking doxycycline for several years. The only new medication was trimethoprim. He was a non-smoker and previously fit and well. 

**Figure 1:**
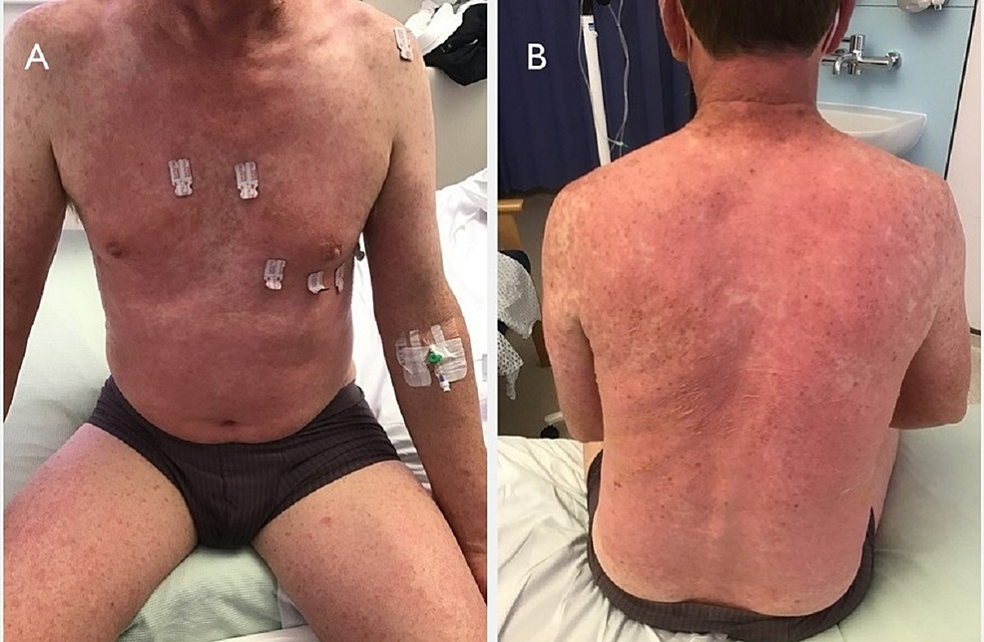
Images taken on admission Generalized, erythematous rash over chest, abdominal wall, and thigh (A), and neck and back (B)

Physical examination on presentation revealed a widespread, erythematous rash over his anterior chest wall, back, and limbs (Figure 1). He was tachycardic with a pulse of 108 beats per minute and pyrexic with a temperature of 39°C. His respiratory rate, blood pressure, and oxygen saturation on room air were normal.

**Figure 2:**
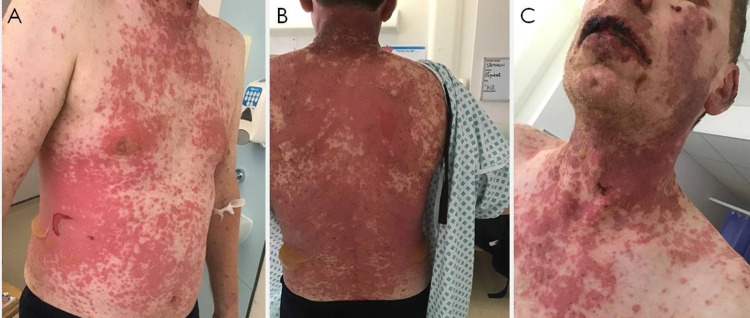
Image of patient with progression to toxic epidermal necrolysis Generalized, erythematous, blistering rash over chest and abdominal wall (A), neck and back (B), and lips and nostrils (C)

The history of trimethoprim use and examination findings such as early ocular involvement, pyrexia and the distribution of lesions supported the early working diagnosis of SJS. However, the condition progressed and the eventual degree of epidermal detachment (over 60% body surface area), bilateral conjunctivitis with subconjunctival hemorrhage, inflammation and sloughing of his nostrils and oral cavity (hemorrhagic mucositis), scrotum, and penile meatus led to the suspicion of TEN by day 4 of admission (Figure 2). His age and the percentage body surface area involved gave him a severity of illness Score for Toxic Epidermal Necrolysis (SCORTEN) of 2, which correlated with a predicted mortality score of 12%.

Investigations

Blood investigations revealed neutrophilia, lymphocytopenia, hyponatremia, and raised inflammatory markers. Renal function and liver function tests were normal (Table 1). His chest X-ray and electrocardiography were normal. Initial blood, urine, and skin cultures revealed no organisms. Serology testing was negative for the hepatitis (B, C) virus, human immunodeficiency virus, varicella-zoster, and the Epstein-Bar virus. Screening for COVID-19 infection was also negative.

**Table 1:** Laboratory tests on admission

Blood test	Patient’s results	Reference range
Sodium (mmol/L)	129	132-145
Potassium (mmol/L)	3.9	3.4-5.1
Creatinine (mmol/L)	97	45-84
Urea (mmol/L)	5.1	2.5-7.8
Lactate (mmol/L)	1.0	0.6-2.5
Glucose (mmol/L)	6.7	4.0-7.0
Total protein (g/L)	67	60-80
Albumin (g/L)	44	35-50
Globulin (g/L)	23	20-35
C-reactive protein (<10)	74	20-35
Hemoglobin (g/L)	163	130-180
White cell count (10^9^/L)	17.1	4.0-11.0
Neutrophil count (10^9^/L)	14.5	1.8-7.7
Lymphocyte count (10^9^/L)	0.9	1.4-4.8

Diagnostic confirmation of TEN was provided two weeks after admission by a punch biopsy taken five days after the initial presentation. Histological examination of the sample revealed total necrosis of the skin and separation of the epidermis, with scanty perivascular inflammation, areas of early re-epithelialization, and squamous metaplasia of the sweat ducts - features consistent with TEN/SJS. Importantly, there was no evidence of fungal infection and immunofluorescence was negative for immunoglobulins G, M, and A, and complement factor C3, ruling out paraneoplastic pemphigoid as a differential.

Treatment

Initial management involved intravenous fluid support, skin care, oral prednisolone, and regular infection screening. However, having identified the progression of his condition from SJS to TEN, treatment was escalated to include intravenous hydrocortisone and prophylactic antibiotics (piperacillin/tazobactam), while continuing with supportive measures such as nutritional support, antipyretics, and emollients. The extensive mucosal involvement in TEN required specialist input from dermatology, ophthalmology, otorhinolaryngology, urology, and maxillofacial specialists to monitor for further deterioration. Early catheterization was advised in anticipation of urological complications, and insertion of a nasogastric feeding tube for adequate nutritional support. Unfortunately, this was not tolerated by the patient, and nutrition was managed orally, with intravenous electrolyte replacement. The patient was nursed in a single-patient room where strict aseptic techniques were observed.

In the acute phase, transfer to the intensive care unit was advised by the dermatologist. However, we encountered this patient during the midst of the COVID-19 pandemic, which led to challenges in attaining the appropriate care setting for this patient. The alternative was to transfer to a specialist burns unit, which was also unsuccessful; however, we sought daily advice from the local burns consultant with regards to managing this severe case of TEN. 

Outcome and follow-up

**Figure 3:**
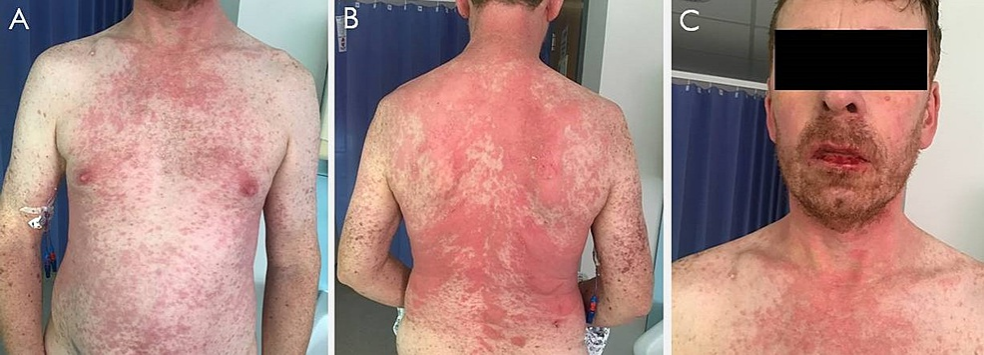
Image of patient recovering from toxic epidermal necrolysis Taken on the day of discharge: chest and abdominal wall (A), neck and back (B), and lips and nostrils (C)

Following nine days of intravenous corticosteroids, antibiotics, and supportive therapy, his skin and mucosal inflammation were under control and no new lesions were identified (Figure 3). The patient was tolerating a soft diet; treatment was stepped down to a reducing course of oral prednisolone, the antibiotics were stopped and he was discharged on day 20 with outpatient follow-up with the relevant medical specialties.

## Discussion

Stevens-Johnson syndrome (SJS) and toxic epidermal necrolysis (TEN) are rare, severe mucocutaneous reactions requiring a full multidisciplinary approach to treatment [1,2]. The erythema, blistering, and sloughing that ensues resembles that of a severe burn and is therefore usually managed as such. TEN is rare, with an incidence rate of 0.4-1.2 per million individuals [3]. Numerous medications have been implicated, including antibiotics, antiepileptic drugs, nonsteroidal anti-inflammatory drugs, ampicillin, allopurinol, corticosteroids, and antiretroviral drugs [4]. A case-controlled study found that the use of trimethoprim-sulfamethoxazole and other sulphonamide antibiotics had the highest risk [5]. The pathogenic mechanisms leading to the development of SJS and TEN are still not fully explained but it is thought that there is a delayed immune response to an antigenic drug-host tissue complex [6].

Stevens-Johnson syndrome/toxic epidermal necrolysis lie on a spectrum of severity, and differentiation between the two is based on the percentage of skin involvement [1,2]. Less than 10% skin involvement is classed as SJS, more than 30% skin involvement is classified as TEN, while 10-30% skin involvement is classed as SJS/TEN overlap. The diagnosis of SJS or TEN is based on clinical and histological evaluation. As our case illustrates, histological confirmation cannot always be provided in the acute phase, therefore placing an emphasis on clinical judgement for early diagnosis.

Differential diagnoses include other severe cutaneous adverse reactions. Infective causes should be ruled out early, and include staphylococcal scalded skin syndrome, toxic shock syndrome, and mycoplasma pneumonia infection, typically causing an atypical SJS eruption. Alternative diagnoses include non-infective causes such as erythema multiforme major (with mucosal involvement), bullous systemic lupus erythematosus, and paraneoplastic pemphigus, the latter of which is reliant on negative immunofluorescence during histological examination [7].

Mortality in SJS/TEN is directly associated with body surface area (BSA). Prognostic assessment within 24 hours of presentation using the SCORTEN scale has been demonstrated to aid evaluation and to predict outcome [8]. The SCORTEN scale is based on the following criteria: age more than 40 years, presence of malignancy, heart rate more than 120 beats per minute, serum glucose more than 14 mmol/L, serum urea more than 10 mmol/L, serum bicarbonate less than 20 mmol/L, and the presence of more than 10% epidermal detachment on admission [8].

The emergency management of patients with SJS or TEN requires a multidisciplinary team approach, withdrawal of the offending drug, and prompt referral to a specialist center where possible for supportive therapy, which forms the mainstay of management [1,2,9].

Despite the abundance of published case reports of drug-induced TEN, especially implicating the combined antibiotic trimethoprim-sulfamethoxazole, published reports implicating trimethoprim alone are scanty in the literature. Trimethoprim is the less toxic portion of the trimethoprim-sulfamethoxazole combination and is commonly used in the United Kingdom for urinary tract infection. We found only three short reports published in the literature implicating trimethoprim as a cause of SJS or TEN. The first reported case was a 74-year-old woman who had severe TEN caused by using trimethoprim alone [10]. The second case involved a patient with a previously documented reaction to trimethoprim, who was receiving it for prophylaxis against infection after ureteric calculus extraction [11]. The third case involved a 31-year-old female who developed Stevens-Johnson syndrome while using trimethoprim alone [12]. We believe that our case may be the fourth report of a severe case of TEN secondary to the use of trimethoprim alone as a causative agent.

Our patient had over 60% skin surface area involvement with a calculated mortality of 12%. This patient required careful measures to prevent secondary bacterial infection, the greatest documented cause of mortality in cases of TEN [8]. This case was uniquely challenging due to the fact the patient presented during the COVID-19 pandemic, limiting our access to bed space on the high dependency and intensive care units.

There are abundant reports and studies involving the use of intravenous corticosteroids, intravenous immunoglobulin, or cyclosporin singularly or in combination for the management of SJS and TEN, none of which conclusively evidence their role in treating patients with SJS/TEN. The current United Kingdom guidelines state “there is no conclusive evidence to demonstrate the beneﬁt of any one of these interventions over conservative management or evidence to demonstrate harm from intravenous immunoglobulin, systemic corticosteroids or cyclosporin in the context of SJS/TEN. The guideline development group considers that, ideally, such interventions should be practised under the supervision of a specialist skin failure multidisciplinary team in the context of a clinical study or a case registry” [2].

## Conclusions

This report details the progression of Stevens-Johnson syndrome to toxic epidermal necrolysis in a 54-year-old man with a history of trimethoprim use. Case reports of TEN implicating trimethoprim alone as an etiological agent are rare in the literature. We hope that this case highlights the importance of early clinical suspicion and management in the context of a similar presentation, early involvement of the wider multidisciplinary team and early supportive measures to minimise progression and the complications of this potentially life threatening condition.
